# Department of Surgery

**Published:** 1900-01

**Authors:** W. E. A. Wyman, L. A. Merillat, J. A. Sloan, Robert Jay

**Affiliations:** Milwaukee, Wis.; M’killip Veterinary College, Chicago; San Francisco, Cal.; West Liberty, Ia.


### DEPARTMENT OF SURGERY.

By W. E. A. Wyman, M.D.V., V.S.,
MILWAUKEE, WIS.

L. A. Merillat, V.S.,
M'KILLIP VETERINARY COLLEGE, CHICAGO.

J. A. Sloan, B.Sc., M.D.V.,

SAN FRANCISCO, CAL.

Dr. Robert Jay, M.D.V.,
WEST LIBERTY, IA.

### The Clinical Diagnosis of Nasal Discharges.

The Department of Surgery of the July and August numbers of the Journal devoted itself to a general survey of nasal discharge and nasal exhalation; the next clinical issue in order shall be the various diseases productive of nasal exhalations and exudates, such as fracture of the nasal, frontal maxillary bones, necrosis, and other diseases of the septum nasi, neoformations, epistaxis, coryza, and diseases of the sinuses and guttural pouches. An endeavor shall be made to deal with the various subjects as practically as possible, viz., from the clinical stand-point—that is, make the history of the case, inspection, palpation, etc., the basis of the discussion.

Since the first two articles on this subject have been received so kindly, it is hoped that the following ones will meet with a similar reception. Before entering upon the diseases themselves a *résumé* of clinical methods of examination may be of some interest.

Examination of the Nose and Nasal Cavity. Inspection of the nostrils reveals various states, infiltrations, and swellings of the skin about the nostrils, as in farcy, morbus maculosus, etc.; of tumors, carcinomata, fibromata, and papillomata are more frequently met; osseous swellings, as in rhachitis, osteoporosis; congenital and traumatic deformities; ulcers and nodules upon the nasal skin and mucous membrane; the latter also exhibits the pustules of the contagious form of stomatitis. The immediate neighborhood of the nostrils may be covered with crusts, resulting from exudations. Thus the amber-colored or dirty yellow-reddish crusts follow the rusty discharge of contagious pneumonia. Any hemorrhagic exudate is liable to give rise to brownish crusts, while the discharge of the streptococcus* distemper, and glanders especially, may result in deposits of yellowish, dirty gray or greenish color. Finally, in discharges of long standing, maceration and destruction of the pigmentary layer occurs, and a fleshy colored streak indicates chronicity and the insulted locality.

The internal inspection of the nasal cavity, as a rule, is done simply with the thumb and index finger, occasionally with a speculum, and, when circumstances warrant it, with the Polansky-Schindelka panelectroscope or rhino-laryngoscope. These last two instruments are practically unknown to the majority of veterinarians, as the price of about $75 is sufficient to create consternation, the entire instrument supply of most men being easily covered by the above sum. At the same time it should be in every college, and the practitioner responsible to a large clientage cannot, except in rare instances, get along without it, as it is the only safe means by which a doubtful diagnosis can be rendered positive. Exploration of the nasal cavity may yield some of the following changes of more or less diagnostic importance.

The most important problem in the art of clinical diagnosis lies not so much in seeing a certain thing, but to interpret correctly that which is detectable. The beginner, therefore, is apt to make diagnostic errors. In this instance the color of the nasal mucous membrane is of interest. An examination with ordinary daylight gives the safest results, as the hyperæmic or anæmic states of the membrane are readily discovered; but even here it must be remembered that the more superior parts of the mucous membrane of the septum nasi and nasal cavity naturally simulate congestion on account of the increased blood-supply of the erectile tissue of that locality, thus appearing darker in color without actually being hyperæmic. In case the speculum is used and artificial light thrown upon the mucous membrane, the healthy reddish color is supplanted by a dark and even yellow-reddish color, while the region of the erectile tissue looks somewhat bluish-red without being congested. The nasal opening of the lachrymal duct, as well as the little indentations representing the openings of mucous glands in the more inferior portion of the mucous membrane of the septum nasi, must not be mistaken for diseased states—ulcerations, for instance. Exudates, the result of a simple inflammatory process involving the nasal mucous membrane, should not be mistaken for pustules or ulcers, and in all doubtful cases may be removed gently with the finger to demonstrate the true origin. Dust covering the mucous membrane, thus hiding the actual state of affairs, may be wiped away with the finger.

In all cases of a suspicious nature the practitioner should conduct the examination with due care, to prevent the possibility of infecting himself.

Color of the Nasal Mucous Membrane. When studying the color of the membrane from a clinical point of view it is well to compare it with the color of some other mucous membrane; for instance, the conjunctiva. The importance of this manifests itself strikingly in the chronic nasal catarrh. Here the nasal mucous membrane's excessive pallor is quite out of proportion to the color exhibited by the other mucous membranes; in fact, the color of the nasal mucous membrane in the chronic nasal catarrh may be termed a leaden hue.

(To be continued.)

